# Effect of wildfire on the prevalence of opioid misuse through anxiety among young adults in the United States: a modeling study

**DOI:** 10.1186/s12889-024-19417-6

**Published:** 2024-07-17

**Authors:** Sigal Maya, Ali Mirzazadeh, James G. Kahn

**Affiliations:** 1 Philip R. Lee Institute for Health Policy Studies, University of California San Francisco, San Francisco, CA USA; 2 Department of Epidemiology and Biostatistics, University of California San Francisco, San Francisco, CA USA

**Keywords:** Wildfire, Substance use, Opioid use, Anxiety

## Abstract

**Background:**

Exposure to climate change events like wildfires can lead to health and mental health problems. While conceptual frameworks have been hypothesized describing the potential relationship between disaster exposure and substance use, the association remains under-researched and unquantified.

**Methods:**

We constructed a quantitative portrayal of one proposed conceptual framework that focuses on the intermediary role of anxiety. We used the Monte Carlo simulation to estimate the impact of wildfire exposure on opioid misuse outcomes through increased anxiety. We searched for and extracted prior empirical evidence on the associations between wildfire anxiety and anxiety-opioid misuse. Three scenarios were devised: in S1 the impact of wildfire on opioid misuse was limited to increasing anxiety incidence; in S2 we also considered the additive role of altered anxiety phenotype; and in S3 we further considered the role of increased opioid-related consequences of pre-existing anxiety due to wildfire exposure.

**Results:**

Models show that the prevalence of opioid misuse post-wildfire may rise to 6.0%-7.2% from a baseline of 5.3%. In S1, the opioid misuse prevalence ratio was 1.12 (95% uncertainty interval [UI]: 1.00 – 1.27). The two exploratory scenarios, with less stringent assumptions, yielded prevalence ratios of 1.23 (95% UI: 1.00 – 1.51) and 1.34 (95% UI: 1.11 – 1.63).

**Conclusions:**

Our modeling study suggests that exposure to wildfires may elevate opioid misuse through increasing anxiety incidence and severity. This can lead to substantial health burdens, possibly beyond the duration of the wildfire event, which may offset recent gains in opioid misuse prevention.

## Introduction

Over the past few years, a growing research base has demonstrated a tremendous impact on mental health related to climate change and extreme weather events [1–8]. The chronic exposure to climate change, such as witnessing changing landscapes, increasing temperatures, and altered weather patterns have been shown to lead to emotional distress broadly, described as eco-anxiety, or solastalgia [7, 8]. On the other hand, acute experiences like wildfires, storms, and heatwaves also lead to poor emotional well-being. Depression, anxiety, post-traumatic stress, and a slew of other mental health outcomes have been found to increase in communities affected by such acute experiences of climate change, in a wide array of settings across the globe [7–10].

Some studies looked at substance use as another health outcome affected by exposure to extreme weather events such as storms, hurricanes, and wildfires [9, 11–13]. Though quantitative evidence is limited, narrative reviews as well as anecdotal evidence have led to the development of several conceptual frameworks describing the relationship between climate change exposures and substance use [14, 15]. Potential mechanisms include changing drug use patterns and access to substances due to altered physical environment, and via poor mental health (e.g., depression, stress, anxiety) in the aftermath of a disaster [11, 16, 17]. Such events may disrupt communities and reduce access to drug treatment services which may further leave individuals vulnerable to poor substance use outcomes [18, 19].

Taken together, the available evidence might be hinting to a brewing syndemic of climate change and substance use. One meta-analysis found that for each 1°C increase in temperature, mortality risk among those with substance-related mental disorders increases 4.6% [20]. In 2021, 60% of heat-associated deaths in Maricopa County, Phoenix, also involved substance use; in 84% of those cases, substance use was a primary cause of death [21]. In Canada, those exposed to the Fort McMurray fire in 2016 had almost three times the prevalence of probable substance use disorders one year later, compared to the national average [22]. Increases in substance misuse were seen similarly in Australian young adults exposed to bushfires [23]. This is concerning for the U.S., where the annual death rate for opioid overdoses is 14.9 per 100,000, and opioid use disorder morbidity and overdose deaths cost over $1 trillion each year [24, 25].

In this modeling analysis, we aimed to quantify the impact of wildfires on opioid misuse in the U.S., through the possible mediating path of anxiety. Shedding light on the magnitude of this association can inform future research on this topic and can help guide public health resources to where they are needed.

## Methods

We quantitatively portrayed the hypothesized pathway from wildfire exposure to increased opioid misuse via increased anxiety incidence and severity [15]. The portrayal utilized epidemiologic calculations of prevalence and risk/odds ratios. Parameters were populated using empirical evidence on the impact of wildfire exposure on anxiety and anxiety on opioid misuse, obtained via literature review [11, 16, 17, 22, 26, 27]. Two variables of interest, the odds ratio for opioid misuse given fire-related anxiety and the prevalence of anxiety given wildfire exposure, were defined as log-normal and beta distributions, respectively. Distribution parameters were determined by visually approximating the histograms of each distribution to the available range of empirical values (Table 1). A Monte Carlo simulation was run with 50,000 iterations to produce probabilistic distributions of two primary outputs: the overall prevalence of opioid misuse after a wildfire event, and the respective prevalence ratio for opioid misuse among those exposed to a wildfire. The model was implemented in R version 4.1.3 and is made available on GitHub [28, 29].

**Table 1:** Model parameters

Parameter	Mean Value	Source	Notes
Prevalence of opioid misuse (calibration target)	5.3%	SAMHSA 2020 [26]	Past-year prevalence of opioid misuse among U.S. youth aged 18–25.
Prevalence of anxiety	6.6%	Global Burden of Disease Study 2019 [27]	Past-year prevalence of anxiety in the U.S. among those aged 20–24.
Anxiety prevalence among those exposed to wildfire	Beta distribution with alpha 10.5 and beta 70	Belleville 2021, [22] authors’ calculation	Likely anxiety (measured via GAD-7) among Fort McMurray residents one year after the wildfire. For comparison, nation-wide prevalence was 3%.
Odds ratio for opioid misuse given fire-related anxiety (Scenario 1)	Log-normal distribution with mean 3.04 and standard deviation 1.07	Moosavi 2019, [11] Ritchie 2021, [16] Agyapong 2018, [17] authors’ calculation	Mean value is the median of 3 studies conducted by the same research group in Fort McMurray, Canada, following the 2016 wildfires. All 3 studies measured anxiety using GAD-7 6–18 months post-fire in different sub-populations.
Risk ratio for opioid misuse among those with anxiety in Scenario 2	5	Hypothetical	Assumes higher risk ratio to reflect pre-disposition to opioid misuse in wildfire-triggered anxiety.
Risk ratio for opioid misuse among those with anxiety in Scenario 3	5	Hypothetical	Assumes higher risk ratio to reflect increased opioid misuse risk among pre-existing anxiety.

The model represents U.S. young adults (ages 18–25), among whom the prevalence of opioid misuse is 40% greater than the national average, and it was calibrated to the overall prevalence of opioid misuse in this group [26]. This was done by manually tuning the parameter for the prevalence of opioid use without anxiety. Calibration was achieved when mean opioid misuse prevalence in the model (with and without anxiety) was 5.3% [26].

**Fig. 1:**
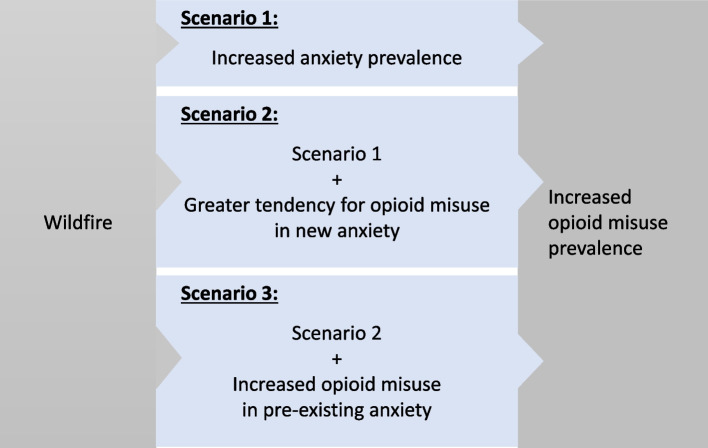
Conceptual framework of the relationship between wildfire experience, anxiety, and opioid misuse

In Scenario 1 (S1), we limited the impact on opioid misuse to the rising incidence of anxiety as an outcome of wildfires. We then devised two additional exploratory scenarios to estimate modified relationships within the conceptual framework (Fig. 1). Scenario 2 (S2) hypothesized that exposure to wildfires would not only increase the prevalence of anxiety but would also be associated with a greater tendency for opioid misuse among those who do develop anxiety. Scenario 3 (S3) additionally included the worsening of pre-existing anxiety following experiencing a wildfire, captured implicitly by applying a higher risk ratio for opioid misuse than in baseline to both pre-existing and incident anxiety.

## Results

**Fig. 2:**
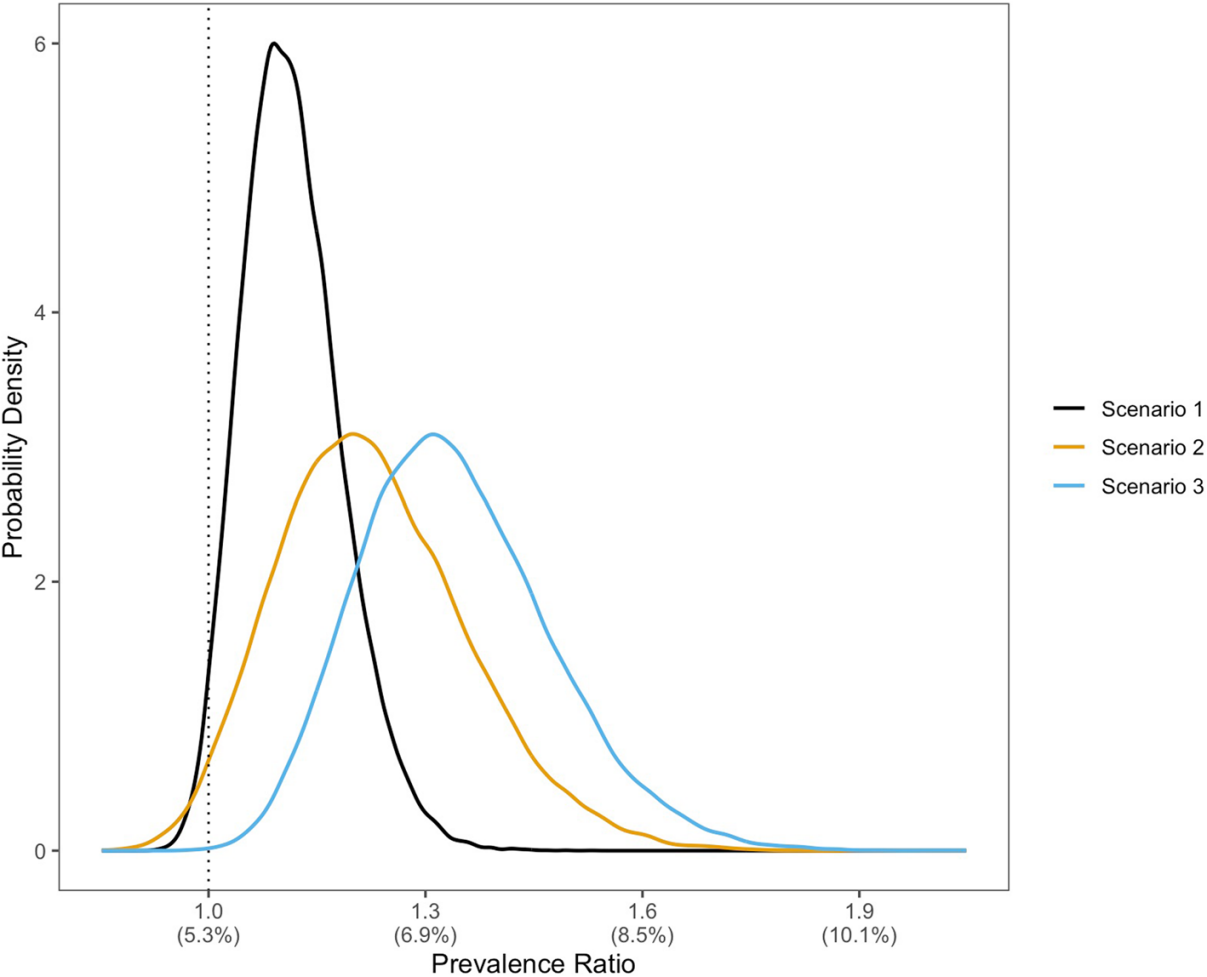
Probability density function for the prevalence ratio for opioid misuse (parentheticals represent the corresponding prevalence value) among those exposed to a wildfire. The area under the curve across a specified range represents the probability of the value of opioid misuse prevalence falling within that range; the total area under each curve is equal to 1. The dotted vertical line reflects a prevalence ratio of 1.00 (and the 2019 prevalence of opioid misuse; 5.3%). Scenario 1 limits the impact of wildfire on opioid misuse to increasing anxiety prevalence. Scenario 2 additionally incorporates a greater tendency for opioid misuse in wildfire-related anxiety. Scenario 3 further incorporates increased opioid-related consequences in pre-existing anxiety following wildfire exposure

The model suggests increasing opioid misuse prevalence following exposure to wildfire (Table 2, Fig. 2). Exposure to a wildfire event led to an increase in the prevalence of anxiety (prevalence ratio 1.98; not shown). In S1, this increase led to an opioid misuse prevalence of 6.0%, reflecting a prevalence ratio (PR) of 1.12 (95% uncertainty interval [UI]: 1.00 – 1.27).

**Table 2:** Mean results of the Monte Carlo simulation (50,000 iterations)

	**Opioid misuse prevalence** **(95% UI)**	**Prevalence ratio for opioid misuse post-wildfire** **(95% UI)**
Scenario 1 (more prevalent anxiety)	6.0% (5.3% – 6.8%)	1.12 (1.00 – 1.27)
Scenario 2 (opioid pre-disposed anxiety)	6.5% (5.3% – 8.1%)	1.23 (1.00 – 1.51)
Scenario 3 (worsening pre-existing anxiety)	7.2% (6.0% – 8.7%)	1.34 (1.11 – 1.63)

*UI* Uncertainty interval

In S2, we assumed exposure to wildfire was not only a predictor of anxiety, but also that wildfire-associated anxiety was more likely to lead to opioid misuse than non-wildfire-related anxiety. Given a hypothetical risk ratio of 5 for opioid misuse given wildfire-induced anxiety, the prevalence of opioid misuse increased further to 6.5% (PR 1.23, 95% UI: 1.00 – 1.51). S3 further considered the impact of wildfire exposure on pre-existing anxiety, wherein wildfires increased the severity of anxiety and made it more likely for those with pre-existing anxiety to misuse opioids. In this scenario, we estimated opioid misuse prevalence as 7.2% (PR 1.34, 95% UI: 1.11 – 1.63). Increasing these hypothetical risk ratios led to linear increases in the calculated opioid misuse prevalence in both scenarios, with a steeper incline in S3 than in S2.

## Discussion

We estimated the magnitude of the effect of wildfire exposure on opioid misuse among young adults in the U.S. Our model showed that exposure to a single wildfire event may substantially elevate the prevalence of opioid misuse, ranging from a 12% to 34% increase. The magnitude of this association depends on the extent to which wildfire exposure alters anxiety incidence and severity.

Opioids present a significant problem in the U.S. with large health and financial burden, all of which can be exacerbated by wildfires. As expected, our findings indicate greater prevalence of anxiety post-wildfire, and in turn greater prevalence of opioid misuse, even in the most limiting assumptions of Scenario 1. This has long-term implications for the health of the U.S. population. While the immediate dangers of experiencing a wildfire are relatively transient, mental health problems like substance use triggered by such an experience may persist for a long time after the wildfire subsides, leading to heightened morbidity and mortality, and associated costs, for many years to follow [22, 30–32]. Consequently, increasing frequency of wildfires and other climate disasters may stunt the gains achieved over the last decade in opioid misuse prevention [26]. This highlights the potential of climate change adaptation and mitigation as primary opioid misuse prevention mechanisms.

The dearth of empirical evidence on wildfire experience and opioid misuse led us to opt for a probabilistic model which incorporates uncertainty around two key parameters that drive results: prevalence of anxiety following wildfire, and the consequent increase in opioid misuse. By defining probability distributions aligned with available evidence for these two parameters, no matter how limited, we were able to produce a range of plausible opioid misuse outcomes which overall indicate a harmful effect from wildfire exposure. Yet, more evidence is still needed to confirm these findings. Evaluations of the counter mechanisms (e.g., potential reductions in substance use due to evacuation to unfamiliar settings, lack of privacy in shelters, greater community support) must also be incorporated into future work in order to gain a comprehensive understanding of the link between wildfire experience and substance use [33, 34].

Our findings should be considered in light of several limitations. First, a key model data point on the relationship between wildfire exposure and anxiety was obtained from a set of studies conducted in Canada, where the government response and community support following the wildfire might have been different than in the U.S. It is generally understood that greater support after a disaster is associated with lessened harmful impacts of that disaster [35]. Second, we relied on a simplified pathway from wildfire to opioid misuse, singling out the role of anxiety alone. In reality, it is likely that more complex mechanisms will be involved, with other intermediary outcomes that may change the prevalence of opioid misuse in either direction, though the overall impact is likely to be negative. Similarly, our portrayal of a single wildfire event may have led us to underestimate health harms. As large-scale natural disasters become more frequent, individuals are at risk for cumulative mental health impacts associated with experiencing subsequent extreme weather events [36]. The effects are likely to be more pronounced in areas at greater risk for wildfires than national averages, however, even indirect exposure to wildfires has been shown to negatively impact mental wellbeing [33]. Lastly, our analysis relied on a single year of substance misuse data from 2019. While this allowed us to circumvent data quality challenges brought on by methodological changes in surveys since then, as well as avoid capturing COVID-19-related differences in substance misuse, it did limit our ability to investigate trends over time.

Despite the limitations, our evaluation suggests that experiencing a wildfire can increase opioid misuse prevalence as much as by a third through increased anxiety incidence and severity, possibly offsetting recent improvements. This warrants further research on opioid misuse outcomes related to wildfires to better understand the impact and whether a downward trajectory in opioid misuse can be maintained given climate change stressors. Future work should focus on identifying and quantifying other possible causal pathways and determine sub-groups who may experience heightened vulnerability to negative mental health impacts, so that timely and appropriate interventions may be designed and implemented.

## Data Availability

All data generated and/or analyzed during this study are included in this published article and its references.
